# Responses of Nitrogen Utilization and Apparent Nitrogen Loss to Different Control Measures in the Wheat and Maize Rotation System

**DOI:** 10.3389/fpls.2017.00160

**Published:** 2017-02-08

**Authors:** Zhengping Peng, Yanan Liu, Yingchun Li, Yahya Abawi, Yanqun Wang, Mingxin Men, Duc-Anh An-Vo

**Affiliations:** ^1^College of Resources and Environmental Sciences, Key Laboratory for Farmland Eco-Environment of Hebei, Agricultural University of HebeiBaoding, China; ^2^Key Laboratory of Agricultural Environment, Institute of Environment and Sustainable Development in Agriculture, Chinese Academy of Agricultural Sciences, Agricultural Ministry of ChinaBeijing, China; ^3^International Centre for Applied Climate Sciences, University of Southern Queensland, ToowoombaQLD, Australia; ^4^Computational Engineering and Science Research Centre, University of Southern Queensland, ToowoombaQLD, Australia

**Keywords:** apparent nitrogen loss, maize, net income, nitrogen efficiency, nitrogen fertilizer measures, wheat

## Abstract

Nitrogen (N) is an essential macronutrient for plant growth and excessive application rates can decrease crop yield and increase N loss into the environment. Field experiments were carried out to understand the effects of N fertilizers on N utilization, crop yield and net income in wheat and maize rotation system of the North China Plain (NCP). Compared to farmers’ N rate (FN), the yield of wheat and maize in reduction N rate by 21–24% based on FN (RN) was improved by 451 kg ha^-1^, N uptakes improved by 17 kg ha^-1^ and net income increased by 1671 CNY ha^-1^, while apparent N loss was reduced by 156 kg ha^-1^. The controlled-release fertilizer with a 20% reduction of RN (CRF80%), a 20% reduction of RN together with dicyandiamide (RN80%+DCD) and a 20% reduction of RN added with nano-carbon (RN80%+NC) all resulted in an improvement in crop yield and decreased the apparent N losses compared to RN. Contrasted with RN80%+NC, the total crop yield in RN80%+DCD improved by 1185 kg ha^-1^, N uptake enhanced by 9 kg ha^-1^ and net income increased by 3929 CNY ha^-1^, while apparent N loss was similar. Therefore, a 37–39% overall decrease in N rate compared to farmers plus the nitrification inhibitor, DCD, was effective N control measure that increased crop yields, enhanced N efficiencies, and improved economic benefits, while mitigating apparent N loss. There is considerable scope for improved N use effieincy in the intensive wheat -maize rotation of the NCP.

## Introduction

In recent decades, the growth of global crop yield has mainly been dependent on increasing application rates of synthetic fertilizers, especially nitrogen (N). Further increases of fertilizer rates are unlikely to be as effective in yield improvement as the N use efficiency sharply declines at higher application rates (Tilman et al., 2002). However, more N fertilizer application is expected to be used in cereal cropping systems to meet the grain demand of 9 billion people in the world by 2050 (Ladha et al., 2005).

North China Plain (NCP) is a major grain producing region in China, accounting for 14% of total grain output in the country from predominant winter-wheat and summer-maize rotation systems. For the winter wheat, in the two high-yield crop regions of Gaocheng and Shenzhou cities in NCP, the farmers’ average N application rate is about 300 kg ha^-1^ (Ma et al., 2015). However, the N application rate of 240 kg ha^-1^ was recommended to satisfy grain yield and N use efficiency of wheat under the limited irrigation (Li et al., 2011).

For the summer maize, the typical farmers’ N rate was more than 500 kg ha^-1^ (Cui et al., 2010), but now it is about 417 kg ha^-1^ (Ma et al., 2015). Results in the winter-wheat and summer-maize rotation system from region-wide experiments demonstrated the economically optimal N rate was from 130 to 160 kg ha^-1^ per crop for the average grain yield of around 5.5 and 6.0 t ha^-1^ in wheat and maize, respectively (Cui et al., 2010). It is evident that N application rates far exceed crop requirements for the maximum wheat and maize yields in NCP. Excessive N fertilizer is lost into environment, not only decreasing N use efficiency (Galloway et al., 2008) but also degrading air (Linquist et al., 2012; Shcherbak et al., 2014), soil (Ju et al., 2006) and water quality (Gao et al., 2015). It is therefore desirable to seek effective ways to increase food production while minimizing N loss and its subsequent environmental damage.

To address this problem, many studies have focused on improving fertilization measures through accurate farmland nutrient management technologies, optimizing fertilizer formulae, researching new fertilizer types and regulating N nutrient supply (Alberto et al., 2014; Rusinamhodzi, 2014; Lin et al., 2015; Liu et al., 2015). The new fertilizer types of controlled-release fertilizer (CRF) can increase N use efficiency and crop yield, but it has a higher cost due to the coating materials (Shaviv, 2001). Recently nitrification inhibitors have also been used as a way to reduce N losses and increase N use efficiencies, but only a few such as dicyandiamide (DCD) and 3,4-dimethylpyrazol phosphate (DMPP) have gained commercial importance for practical use. Both DCD and DMPP were found to increase crop yield and N use efficiency and reduce N_2_O emission from the wheat-maize cropping system (Liu et al., 2013). Nano carbon (NC) has small size, surface effect, adsorption and other features and has been found to improve wetland rice yield by 10.2% and N agronomic efficiency by 40.1% while minimizing N losses when added into urea compared to urea application alone (Wang et al., 2010). However, it is not clear if the beneficial effects of NC would be achieved for the irrigated, dryland wheat and maize cropping systems in NCP. Besides, although the effects of CRF, DCD, and NC have been demonstrated, respectively, by many studies as described above, the relative effectiveness of these measures was seldom reported.

The application of excessive N fertilizer rates can be avoided by taking into account specific-site conditions affecting residual soil N, such as crop management practices and rotation systems. Farmers’ N fertilizer application rates can be adjusted accounting for actual crop needs through accounting for the amount of N left by the previous crop. As a result, the winter-wheat and summer-maize rotation system in NCP was chosen for this study. The overall aims are to (1) determine a reduction in N fertilizer compared to the farmers’ N rate based on local crop planting history and soil N content, without affecting wheat and maize yields; (2) evaluate the effects of lower N rates on wheat and maize yield, N efficiency, net income and apparent N loss using alternative fertilizer formulation; and (3) confirm new fertilizer management strategies to secure wheat and maize production with decreasing N fertilizer rates, cost and loss into the environment.

## Materials and Methods

### Site Characteristics

The experimental site (N 38°49′, E 115°26′, China) is situated within the Science and Technology Park of Hebei Agricultural University, China. The area has a temperate monsoon climate with cold dry winter and hot rainy summer. Annual average temperature is about 12°C, annual average sunshine hours total 2660 h, and the average frost-free period is 210 days. Soil collected from the plow layers (0–20 cm) in the field and determined by the conventional chemical analysis method (Shi, 1976), is typically classified as fluvo-aquic cinnamon soil with pH 8.7, total N 1.26 g kg^-1^, alkali-hydrolyzable-N 76.5 mg kg^-1^, available-P 15.1 mg kg^-1^, available-K 125.4 mg kg^-1^, and organic matter of 12.0 g kg^-1^. This experiment was conducted where the winter-wheat and summer-maize rotation system has been practiced for more than 30 years. Winter wheat cultivar was Henong 5274 and the maize cultivar was Zhengdan 958.

### Field Experiment Design

Measurements were carried out from the wheat season of 2013 followed by the maize season and repeated in 2014. Seven treatments were set up as CK (zero N), FN (farmers’ N rate), RN (reduction N rate by 21–24% based on N carry over from the previous season and on crop demand), CRF (CRF with the same N rate as RN), CRF80% (CRF with a 20% reduction of RN), RN80%+DCD (a 20% reduction of RN together with DCD, DCD is applied at 0.5% of N fertilizer with a N content of 69.5%), RN80%+NC (20% reduction N rate of RN added with NC, NC is applied at 0.3% of the total fertilizer rates). The same treatment names but different N fertilizer rates were used for wheat and maize. Details of different treatments in wheat and maize seasons are presented in **Table 1**. The CRF used for wheat is coated by a single layer of resin and has N content of 43%, and by double layers of resin and sulfur for N content of 38% when applied to maize.

**Table 1:** Fertilizer application rates of treatments in wheat and maize seasons (kg ha^-1^).

Treatments	Code	Wheat			Maize		
			
		N	P_2_O_5_	K_2_O	N	P_2_O_5_	K_2_O
Zero N	CK	0	120	150	0	90	150
Farmers’ N rate	FN	285	120	150	392	90	150
Reduction N rate based on N carry over from the previous season and on crop demand	RN	225	120	150	300	90	150
Controlled-release fertilizer with the same N rate as RN	CRF	225	120	150	300	90	150
Controlled-release fertilizer with a 20% reduction of RN	CRF80%	180	120	150	240	90	150
20% reduction of RN added with DCD	RN80%+DCD	180	120	150	240	90	150
20% reduction of RN added with NC	RN80%+NC	180	120	150	240	90	150


*Abbreviations of CK, FN, RN, CRF, DCD and NC respectively represent control, farmers’ N rate, reduction N rate, controlled-release fertilizer, dicyandiamide, and nano carbon.*

The annual application rates of P and K fertilizers in the forms of calcium superphosphate (P_2_O_5_ 16%) and potassium sulfate (K_2_O 60%) were kept identical for seven treatments at the same crop season. In the wheat season, all the P and K fertilizers and 50% of N fertilizer were applied as sowing, and the remaining 50% of N fertilizer was applied as topdressing close to the greening stage coinciding with the first spring irrigation. In the maize season, all the P and K fertilizers and 40% of N fertilizers were applied as sowing, and the remaining 60% of N fertilizer was topdressed close to the jointing stage. Dicyandiamide and NC were applied as both basal fertilizer and by topdressing. Plot size was about 30 m^2^. Seven treatments with three replicates were arranged in a randomized complete block design.

All wheat basal fertilizers in each treatment were mixed together and broadcasted on the soil surface by hand before planting, followed by land preparation and sowing with machine. Wheat was sown with a seed rate of 187.5 kg ha^-1^. After wheat harvest, the straw was directly returned into fields. Similarly, all basal fertilizers in each treatment for maize were mixed together and broadcasted on the soil surface by hand, followed by land preparation and sowing seed rate of 37.5 kg ha^-1^ with machine. Except for the fertilization, other farming practices of all experiment plots were consistent with the farmers’ management methods.

### Soil Sampling and Measurements

Soil samples from 0–20, 20–40, and 40–60 cm (three random core samples from each plot thoroughly mixed together for each layer) were taken at wheat physiological stages of seeding, greening, jointing, grain filling and maturity and maize growth stages of jointing, silking, grain filling, and maturity. Soil samples were placed in plastic bags and transported to the laboratory, where moist soil was sieved (2 mm), homogenized and stored at 4°C for analyses of NO_3_^-^-N concentrations. Soil water content was determined gravimetrically after drying for 24 h at 105°C. Soil NO_3_^-^-N was extracted with 1 mol KCl L^-1^ with a soil-solution ration of 1:10 and analyzed by continuous flow analysis technique.

### Plant Harvest

For wheat harvest, 1 m long double rows were cut for each plot. Effective spike numbers were counted from 50 selected spikes. All harvested samples were threshed and grain moisture was measured with a crop moisture meter (PM-8188). Grain yield was standardized at 12.5% moisture content. Three samples were weighed to get the average 1000-grain weight for each plot. Straw and grain from some plants were killed at 105°C for 30 min, then dried at 75°C to constant weight and ground into powder to test the total N content using a modified Kjeldahl digestion method (Nelson and Sommers, 1973).

At maize harvest, two representative plants were cut and separated into stem with leaves, grain and ear axis. All aboveground parts of the plants were killed, dried, ground into powder and the total N content determined using the same method as for wheat. The rest of maize ears in each plot were collected and twenty ears with the average weight were chosen to measure bald tip length, ear diameter, ear length, grain number per ear and the 100-grain weight. All maize grains were machine threshed and the grain moisture content was determined using the same method as for wheat grain. Grain yields of maize were standardized at 14% water content.

### Nitrogen Efficiency and N Balance Calculation

Crop N efficiency was described by N uptake content and N recovery efficiency (NRE). Using the N element balance principle, N balance status from pre-planting and post-harvest of wheat and maize were calculated (Min et al., 2011). Nitrogen total input was composed of N fertilizer, soil residual N pre-planting, N deposition from dry and wet atmosphere, pre-crop straw return N and mineralized soil N at planting. Nitrogen total output comprised N uptake of crop, soil N post-harvest and apparent N loss. Soil N in 0–60 cm depth was studied. Atmospheric N deposition was derived from Liu et al. (2010). The detailed calculation methods were as follows:

NRE(%)=(Plant N content with N fertilizer-Plant N content without N fertilizer)/N fertilizer rate×100

Mineralized N (kg/ha)=Crop N uptake in CK + Soil residual N post-harvest in CK-Soil N pre-planting-N deposition from atmosphere-Pre-crop straw return N

Apparent N loss(kg/ha)=Total N input-N uptake of crop-Soil residual N post-harvest

### Net Income Analyses

In the economic benefit analysis, production costs included fertilizer and other field management costs. Fertilizer prices in Chinese Yuan (CNY: 1 USD = 6.75 CNY in November 2016) were N 3.9 CNY kg^-1^, P_2_O_5_ 5.65 CNY kg^-1^, K_2_O 6.5 CNY kg^-1^, DCD 20 CNY kg^-1^, NC 260 CNY kg^-1^, CRF in wheat 4.0 CNY kg^-1^, and CRF in maize 6.5 CNY kg^-1^. Field management costs were composed of seed, fertilization and the associated labor costs. Seed costs for wheat and maize were 750 CNY ha^-1^ and 600 CNY ha^-1^, respectively. Labor cost for fertilization was 1500 CNY ha^-1^ per application (zero in CK, once in CRF and CRF80%, and twice in other N treatments). Irrigation, insecticide spray, mechanical sowing and reaping costs in wheat and maize were 2672.3 and 2656.5 CNY ha^-1^, respectively. Grain prices of wheat and maize at harvest were 2.0 and 2.2 CNY kg^-1^, respectively.

### Statistical Analysis

Grain yields of wheat and maize, yield components and the total N content in plant were obtained from three replicates of each treatment at harvest. The data were statistically analyzed using one-way ANOVA, and the mean values were compared by Duncan’s multiple range test at the 5% levels using the SAS (Cary, 2008). Differences between treatments in all tables were tested at the *P* ≤ 0.05.

## Results

### Crop Yield and Yield Components

As shown in **Table 2**, the application of N fertilizer significantly increased the effective spikes, grain number per spike, 1000-grain weight and grain yield of wheat as compared with CK. Relative to FN, the effective spikes and grain number per spike was not significant in other treatments, but 1000-grain weight in RN80%+DCD and grain yields in CRF80%, RN80%+DCD and RN80%+NC were significantly increased. Grain yields in CRF80%, RN80%+DCD and RN80%+NC with N fertilizer rates of 180 kg ha^-1^ were 16% more than that in RN with N fertilizer rate of 225 kg ha^-1^. There was no significant difference among CRF80%, RN80%+DCD and RN80%+NC. Grain yield in CRF80% was higher by 6.3% as compared to CRF, despite a 20% reduction and could be further improved by additional control measures such as RN80%+DCD and RN80%+NC.

**Table 2:** Wheat yield and yield components with different N control measures.

Treatments	Nitrogen fertilizer (kg ha^-1^)	Effective spike (10 thousands ha^-1^)	Grain number per spike	1000-grain weight (g)	Grain yield (kgha^-1^)
CK	0	426b	32.1b	32.2c	5173e
FN	285	663a	42.0a	36.8b	8469cd
RN	225	614a	40.7a	36.6b	8033d
CRF	225	712a	42.0a	38.7ab	8771bc
CRF80%	180	724a	41.6a	38.6ab	9325ab
RN80%+DCD	180	702a	40.4a	41.0a	9507a
RN80%+NC	180	759a	40.8a	38.0ab	9473a


*Treatments of CK, FN, RN, CRF, CRF80%, RN80%+DCD and RN80%+NC represent the corresponding treatments as described in **Table 1**. Means followed by different letters in the same column indicate significant differences between treatments according to one-way ANOVA followed by Duncan’s multiple range test (*P* ≤ 0.05).*

For maize, ear diameter, ear length, grain number per ear, 100-grain weight and grain yield in all N fertilizers treatments were higher than those of CK, especially grain number per ear and grain yield (**Table 3**), while the bald tip length was significantly decreased by different N fertilizer treatments. Compared with FN, grain yields in RN, CRF80% and RN80%+DCD increased by 11.1, 17.1, and 14.8%, respectively. Grain yields in CRF80% and RN80%+DCD were about 4% more than that in RN. Among the N fertilizer rates of 240 kg ha^-1^, grain yields in CRF80% and RN80%+DCD were higher than that in RN80%+NC and there was no significant difference between CRF80% and RN80%+DCD. Grain yield in CRF80% was 15.5% more than CRF. These results illustrate that maize grain yield was not negatively impacted by the reduction in N rate relative to FN and could be enhanced through additional control measures such as CRF80% and RN80%+DCD.

**Table 3:** Maize yield and yield components with different N control measures.

Treatments	Nitrogen fertilizer (kg ha^-1^)	Bald tip length (cm)	Ear diameter (cm)	Ear length (cm)	Grain number per ear	100-grain weight (g)	Grain yield (kg ha^-1^)
CK	0	1.08a	4.48b	14.2b	449b	27.0a	6849c
FN	392	0.62b	4.62ab	15.2ab	507a	27.9a	8009b
RN	300	0.74b	4.68a	15.7a	515a	28.0a	8896ab
CRF	300	0.62b	4.66a	15.1ab	511a	29.2a	8122b
CRF80%	240	0.66b	4.63ab	15.9a	538a	29.8a	9381a
RN80%+DCD	240	0.72b	4.66a	15.2ab	515a	29.9a	9196ab
RN80%+NC	240	0.83b	4.63ab	15.1ab	519a	27.6a	8045b


*Treatments of CK, FN, RN, CRF, CRF80%, RN80%+DCD and RN80%+NC represent the corresponding treatments as described in **Table 1**. Means followed by different letters in the same column indicate significant differences between treatments according to one-way ANOVA followed by Duncan’s multiple range test (*P* ≤ 0.05).*

### Crop N Effiencies

Nitrogen uptakes and recovery efficiencies of wheat and maize in different N control measures are described in **Table 4**. Nitrogen uptakes of wheat and maize in all N fertilizers were significantly increased by 24.1–34.2 and 28.4–36.1%, respectively, compared with those in CK. There were no significant differences among all N fertilizers. NREs of RN in wheat and maize were enhanced by 8.0 and 6.8% in contrast with FN. Compared to RN, NREs of CRF80%, 80%+DCD and RN80%+NC in wheat and maize were significantly increased by 7.6–13.3 and 4.2–7.4%, respectively. NREs of RN80%+DCD in wheat and maize were the highest among all N fertilizers. It is clear that the farmers’ N fertilizer rates are high and a reduction in RN based on N carry over from the previous season and on crop demand, coupled with different N control measures, could increase crop N efficiency.

**Table 4:** Crop N uptake and recovery efficiencies with different N control measures.

Treatments	Wheat			Maize		
		
	Nitrogen fertilizer (kg ha^-1^)	Nitrogen uptake (kg ha^-1^)	Nitrogen recovery efficiency (%)^a^	Nitrogen fertilizer (kg ha^-1^)	Nitrogen uptake (kg ha^-1^)	Nitrogen recovery efficiency (%)
CK	0	199b	-	0	183b	-
FN	285	247a	16.7e	392	235a	13.5d
RN	225	255a	24.7d	300	244a	20.3bc
CRF	225	265a	29.2c	300	239a	18.9c
CRF80%	180	257a	32.3bc	240	241a	24.5ab
RN80%+DCD	180	267a	38.0a	240	249a	27.7a
RN80%+NC	180	264a	35.9ab	240	243a	25.3a


*^a^NRE (%) = (Plant N content with N fertilizer-Plant N content without N fertilizer)/N fertilizer rate × 100. Treatments of CK, FN, RN, CRF, CRF80%, RN80%+DCD and RN80%+NC represent the corresponding treatments as described in **Table 1**. Means followed by different letters in the same column for wheat or maize indicate significant differences between treatments according to one-way ANOVA followed by Duncan’s multiple range test (*P* ≤ 0.05).*

### Net Income Analyses

The economic benefit analyses with different N control measures are shown in **Table 5**. Compared with FN, the net incomes in CRF, CRF80%, RN80%+DCD and RN80%+NC of wheat increased by 1123–2648 CNY ha^-1^ (14.5–34.2% improvement), while the net incomes in RN, CRF80% and RN80%+DCD for maize enhanced by 1941–2962 CNY ha^-1^ (23.2–35.5% improvement). Net incomes of CRF80%, RN80%+DCD, and RN80%+NC in wheat were all higher than that of RN, but for maize only net income of RN80%+DCD was higher.

**Table 5:** Crop economic benefit analyses with different N control measures.

Crop	Treatments	Nirogen fertilizer (kg ha^-1^)	Output value^a^ (CNY ha^-1^)	Fertilizer cost^b^ (CNY ha^-1^)	Other field management cost (CNY ha^-1^)	Net income^c^ (CNY ha^-1^)
Wheat	FN	285	16939c	2765	6422	7752d
	RN	225	16067d	2531	6422	7114c
	CRF	225	17543b	3746	4922	8875b
	CRF80%	180	18649a	3327	4922	10400a
	RN80%+DCD	180	19014a	2626	6422	9966a
	RN80%+NC	180	18945a	3441	6422	9082b
Maize	FN	392	17619b	3010	6257	8352c
	RN	300	19572ab	2654	6257	10661b
	CRF	300	17869b	6615	4757	6497d
	CRF80%	240	20639a	5589	4757	10293b
	RN80%+DCD	240	20231ab	2660	6257	11314a
	RN80%+NC	240	17700b	3174	6257	8269c


*^a^Output value (CNY/ha) = Grain yield × Grain price. ^b^Production costs include fertilizer and other field management costs. Field management costs are composed of seed, fertilization and the associated labor costs. Labor costs include fertilization, irrigation, insecticide spray, mechanical sowing and reaping costs in wheat and maize. ^c^ Net income (CNY/ha) = Output value - Fertilizer cost - Other field management costs. Treatments of FN, RN, CRF, CRF80%, RN80%+DCD and RN80%+NC represent the corresponding treatments as described in **Table 1**. Means followed by different letters in the same column for wheat or maize indicate significant differences between treatments according to one-way ANOVA followed by Duncan’s multiple range test (*P* ≤ 0.05).*

Among all treatments in wheat, net income was the highest in CRF80% and second highest in RN80%+DCD with values of 10400 and 9966 CNY ha^-1^, respectively(34.2 and 28.6% improvement compared with FN). Although the output value in RN80%+DCD was the highest (19014 CNY ha^-1^), it required two application of fertilizer as basal and topdressing resulting in higher labor cost and lower net income. In CRF80% treatment with single resin coating the fertilizer cost was higher (701 CNY ha^-1^) but other field management costs decreased by 1500 CNY ha^-1^ resulting in a net income increase of 434 CNY ha^-1^. Therefore, CRF80% was the best treatment resulting in higher net income.

For maize, among all treatments net income was the highest in RN80%+DCD (11314 CNY ha^-1^) and lowest in CRF (6497 CNY ha^-1^). Output value in CRF80% was the highest (20639 CNY ha^-1^) with only one fertilizer application cost. However, the high fertilizer cost of CRF80% (2929 CNY ha^-1^) resulted in lower net income (1021 CNY ha^-1^) than in RN80%+DCD. Therefore, RN80%+DCD was the best resulting in improved grain yield and higher net income.

### Variation of Soil NO_3_^-^-N Content

**FIGURE 1:**
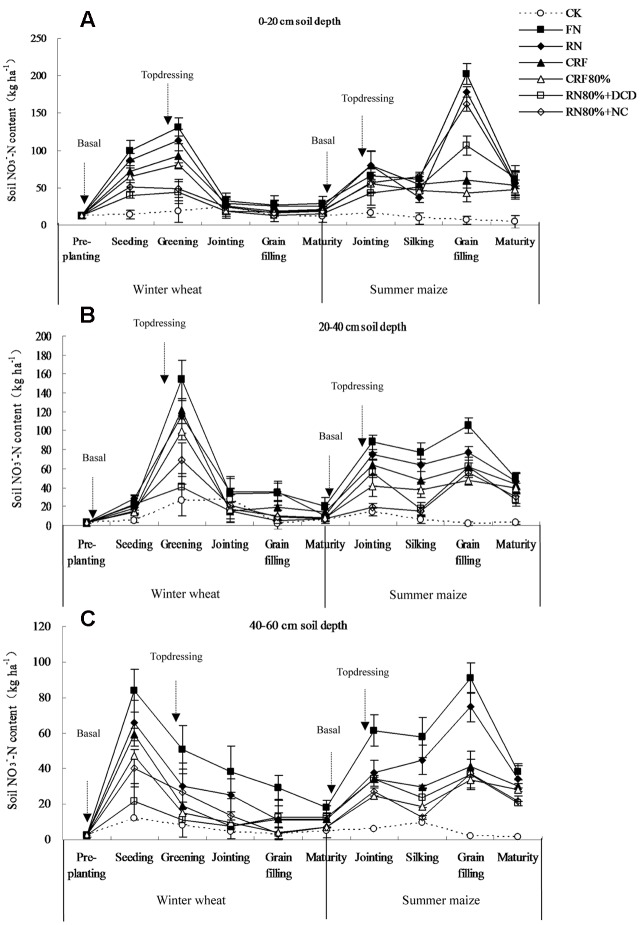
**Temporal and spatial variations of soil NO_3_^-^-N content with different N control measures.** **(A)** 0–20 cm soil depth, **(B)** 20–40 cm soil depth, and **(C)** 40–60 cm soil depth. The bars denote standard deviation of the three replicates. Treatments of CK, FN, RN, CRF, CRF80%, RN80%+DCD, and RN80%+NC represent the corresponding treatments as described in **Table 1**. Basal and topdressing are the times of fertilization. Treatments of FN, RN, RN80%+DCD and RN80%+NC are topdressed, while CK, CRF, and CRF80% not.

During the wheat and maize growth stages, soil NO_3_^-^-N contents varied depending on the N control measures applied (**Figure 1**). Soil NO_3_^-^-N of CK in different soil layers was the lowest, about 10 kg ha^-1^.

With wheat plant growth in different soil layers, soil NO_3_^-^-N among all the treatments increased at the beginning, then declined sharply between greening and jointing stage before settling at a steady level. The peak values of soil NO_3_^-^-N in 0–20 and 20–40 cm soil depths were both at the greening stage, while in 40–60 cm soil depth it was at the seeding stage. With the maize plant growth in different soil layers, soil NO_3_^-^-N in all N fertilizer treatments rose to the jointing stage, then reduced before rapidly rising again from silking to grain filling stages followed by further sharp decline.

Under the same stage of crop and among all the treatments, soil NO_3_^-^-N in FN was higher than that in RN, CRF was more than CRF80%, while CRF80%, RN80%+DCD and RN80%+NC were higher than RN.

### Soil N Balance Analyses

With the decrease of N application rate, soil N at post-harvest and apparent N loss were both reduced in wheat and maize seasons (**Table 6**). In the wheat season, the N fertilizer rate and apparent N loss of RN were lower by 60 and 42.7 kg ha^-1^ than those in FN, and apparent N loss of RN was higher by 12.1 kg ha^-1^ compared to CRF. Nitrogen fertilizer application rate and apparent N loss in CRF were higher by 45 and 27.8 kg ha^-1^ in comparison with CRF80%. Relative to RN, N fertilizer application rates in CRF80%, RN80%+DCD and RN80%+NC decreased by 45 kg ha^-1^ and apparent N losses reduced by 39.9–50.2 kg ha^-1^. Under the N fertilizer rates of 180 kg ha^-1^, apparent N loss in RN80%+DCD was the lowest.

**Table 6:** Nitrogen balance in the soil depth of 0–60 cm with different N control measures.

Crop	Treatments	Nitrogen total input (kg ha^-1^)^a^						Nitrogen total output (kg ha^-1^)^b^		
			
		Fertilizer N	Soil residual N pre-planting	Pre-wheat straw N^c^	Deposition N^d^	Mineralized N at planting^e^	Total N input	Crop uptake	Soil residual N post- harvest	Apparent N loss^f^
Wheat	CK	0	16.4	77.2	12.8	115	221.4	199	22.4	0
	FN	285	16.4	77.2	12.8	115	506.4	247	67.3	192.1
	RN	225	16.4	77.2	12.8	115	446.4	255	42.0	149.4
	CRF	225	16.4	77.2	12.8	115	446.4	265	44.1	137.3
	CRF80%	180	16.4	77.2	12.8	115	401.4	257	34.9	109.5
	RN80%+DCD	180	16.4	77.2	12.8	115	401.4	267	35.2	99.2
	RN80%+NC	180	16.4	77.2	12.8	115	401.4	264	32.8	104.6
Maize	CK	0	22.4	27.9	15.0	126.1	191.4	183	8.4	0
	FN	392	67.3	40.3	15.0	126.1	640.7	235	150.0	255.7
	RN	300	42.0	41.8	15.0	126.1	524.9	244	138.2	142.7
	CRF	300	44.1	45.5	15.0	126.1	530.7	239	150.6	141.1
	CRF80%	240	34.9	57.9	15.0	126.1	473.9	241	115.0	117.9
	RN80%+DCD	240	35.2	46.3	15.0	126.1	462.6	249	112.1	101.5
	RN80%+NC	240	32.8	36.0	15.0	126.1	449.9	243	107.2	99.7


*^a^Nitrogen balance status from pre-planting and post-harvest of wheat and maize are calculated. Nitrogen total input is composed of N fertilizer, soil N pre-planting, pre-crop straw return N, N deposition from dry and wet atmosphere and mineralized N at planting. ^b^Nitrogen total output comprises N uptake of crop, soil N post-harvest and apparent N loss. ^c^In wheat season, the pre-maize straw N is from the maize result of FN in this paper. ^d^Atmospheric N deposition is derived from Liu et al. (2010). ^e^Mineralized N (kg/ha) = N crop uptake in CK + Soil residual N post-harvest in CK - Soil residual N pre-planting - N deposition from atmosphere - Pre-crop straw return N. ^f^Apparent N loss (kg/ha) = Total N input - N uptake of crop - Soil residual N post-harvest. Treatments of CK, FN, RN, CRF, CRF80%, RN80%+DCD and RN80%+NC represent the corresponding treatments as described in **Table 1**.*

For the maize, N fertilizer application rate, N total input and apparent N loss of RN were lower by 92, 115.8, and 113 kg ha^-1^ relative to those in FN. Apparent N loss in RN was slightly higher than that in CRF with the same N rates. Nitrogen fertilizer rate, N total input and apparent N loss in CRF were higher by 60, 56.8, and 23.2 kg ha^-1^ than those in CRF80%. Compared with RN, N fertilizer rates in CRF80%, RN80%+DCD and RN80%+NC decreased by 60 kg ha^-1^, total N input reduced by 51–75 kg ha^-1^ and their apparent N losses reduced by 24.8–43 kg ha^-1^. Under the N fertilizer rates of 240 kg ha^-1^, apparent N loss in RN80%+DCD was higher than that in RN80%+NC and lower than that in CRF80%.

These results showed RN80%+DCD and RN80%+NC could improve the wheat and maize yields and reduce N loss compared to FN and RN.

## Discussion

### Nitrogen Reduction Efficiency

Grain production has grown dramatically mainly due to increased application of N fertilizer. In NCP, under the temperate semi-humid farming conditions, urea applied to the soil is transformed into nitrate within 1–2 weeks. Together with the high application rate of N fertilizer and the crop’s low N recovery there is substantial accumulation of residual nitrate in the soil profile (Ju et al., 2007). In practice the N fertilizer rate should be adjusted according to the residual nitrate and the local conditions. In our experiment, the N rates in FN were 285 and 392 kg ha^-1^, respectively in wheat and maize, while the rates in RN were 225 and 300 kg ha^-1^ according to the crop yield, soil fertility, irrigation conditions, pre-plant straw return and many years of field experiment results in NCP. Grain yields of wheat and maize had no significant difference between FN and RN (**Tables 2** and **3**). NREs of RN in wheat and maize were both enhanced, relative to FN (**Table 4**). Farmers’ N had excessive N fertilizer rates without additional crop demand leading to more apparent N loss in wheat and maize compared to RN (**Table 6**). Under the same crop growth stage and different soil depths, soil NO_3_^-^-N in RN was lower as compared to FN (**Figure 1**).

From the whole rotation system, compared to FN, the total crop yield of wheat and maize in RN was unchanged, N uptakes improved by 17 kg ha^-1^, net income increased by 1671 CNY ha^-1^, while apparent N loss reduced by 156 kg ha^-1^ (**Table 7**). As a result, the proposed reduction N application rate does not negatively affect the wheat and maize total grain yields, but improves N efficiency and net income, decreases the production cost and N loss into agricultural soil.

**Table 7:** Total analyses of winter wheat and summer maize with different N control measures.

Treatments	Total yield (kg ha^-1^)	Total N uptake (kg ha^-1^)	Total net income (CNY ha^-1^)	Total apparent N loss (kg ha^-1^)
FN	16478c	482c	16104c	448a
RN	16929c	499b	17775b	292b
CRF	16893c	504b	15372d	278b
CRF80%	18706a	498b	20693a	227c
RN80%+DCD	18703a	516a	21280a	201d
RN80%+NC	17518b	507ab	17351b	204cd


*Total yield, N uptake, net income, apparent N loss are the sum of wheat and maize. Treatments of CK, FN, RN, CRF, CRF80%, RN80%+DCD and RN80%+NC represent the corresponding treatments as described in **Table 1**. Means followed by different letters in the same column indicate significant differences between treatments according to one-way ANOVA followed by Duncan’s multiple range test (*P* ≤ 0.05).*

### Effects of Lower N Rates with CRF

Some N control measures can further reduce N rate without influencing the crop growth. An example is CRF commonly coated by sulfur, polyethylene, polyvinyl chloride, latex, oil, attapulgite, and other synthetic substances (Guan et al., 2014). Forms of CRF significantly improved yields of wheat (Yang et al., 2011), corn (Ding et al., 2011) and other crops reducing the amount of fertilizer and the labor costs. Despite the environmental and agronomic benefits offered by CRF, their practical use in agriculture is still very limited by high price associated with the complex coating technologies.

In our study, the cost of CRF wrapped by single layer of resin in wheat was less than that wrapped by double layers of resin and sulfur for use with maize. Under the same N fertilizer rate, compared with RN, grain yield in CRF wheat was higher by 9.2% (**Table 2**), but maize yield was 8.7% lower (**Table 3**). This conflicting result might be due to different climate conditions and coated materials for wheat and maize (Yu et al., 2010). NRE in CRF as compared to RN improved by 4.5% for wheat (**Table 4**) but no difference was found in maize. The net income in wheat increased, while in maize reduced (**Table 5**), and apparent N losses decreased both in wheat and maize (**Table 6**). From the total rotation system, relative to RN, the total crop yield and N uptake in CRF were similar, while total net income decreased by 2403 CNY ha^-1^ (**Table 7**). Yields, N uptakes, NREs, net income for both wheat and maize in CRF80% were all higher and apparent N losses lower than those in CRF and RN (**Tables 2–7**).

Under the same crop growth stage and different soil depths, soil NO_3_^-^-N in CRF was lower than that in RN, but higher than that in CRF80% (**Figure 1**). These results indicate that CRF with rational N rates can improve crop yield and N efficiencies, reduce the amount of fertilizer and the labor costs, reduce N leaching losses into environment. However, because of its expensive coating cost, the net income was not high, especially for maize. Possibly, improved technologies producing cheaper CRF, together with optimal design of fertilizer composition and coatings can achieve better effects in future.

### Effects of Lower N Rates with NC and DCD

The total yield and net incomes of wheat and maize in CRF80% were similar with that in RN80%+DCD, but total yield was 6.8% higher (1188 kg ha^-1^) than in RN80%+NC. Compared to RN, total N uptake of wheat and maize in 80%+DCD improved by 17 kg ha^-1^. For both crops total apparent N loss in RN80%+DCD was the lowest (**Table 7**). The higher N uptake (**Table 3**) and plant recovery efficiency (**Table 4**) probably explain the lower N loss in RN80%+DCD.

Under normal conditions, free NH_4_^+^ does not exist and most of the inorganic N occurs as NO_3_^-^ in the soil (Liu et al., 2013). Nitrogen application rates had little affect on NH_4_^+^-N content in the 0–100 cm soil profiles, but led to excess NO_3_^-^-N accumulated in the 0–100 cm soil profile (Fang et al., 2006). In our experiment, the NH_4_^+^-N content in the 0–60 cm soil profiles was very little, so only the soil NO_3_^-^-N content was studied. Application of DCD shifts the primary form of soil inorganic N from NO_3_^-^ to NH_4_^+^ (Weiske et al., 2001) and may increase the ratio of NH_4_^+^/NO_3_^-^. Previous results also have indicated that wheat and maize produce higher yields and dry matter when supplied with mixtures of NH_4_^+^ and NO_3_^-^ than only with one of them (Manuel et al., 1992; Smiciklas and Below, 1992). Once applied to soil, DCD is susceptible to biodegradation but was reported to increase wheat and rice yields (Bhatia et al., 2010), although a slight reduction in crop yield was reported by Mahmood et al. (2011).

As has been reported by Wang et al. (2010) NC could increase rice yield and minimize N loss in wetland, results from the present study verified that these effects of NC (RN80%+NC) could be achieved in the irrigated dryland wheat and maize production as well. Moreover, comparing to RN80%+NC, the crop total yield, N utilization and net income in RN80%+DCD were further enhanced while the total apparent N loss was unchanged (**Table 7**).

Therefore, RN80%+DCD could be proposed as an effective N control measure by increasing yields, enhancing N utilization, boosting economic benefit and mitigating N loss although N loss was still substantial (**Table 7**). Consequently, further studies on measures to decrease N loss are warranted.

## Conclusion

Increasing crop productivity and improving N efficiency are not only related to maximizing the biological potential of crops, but also necessary for the sustainability of China’s agricultural production. We found a reduction in N application rate based on N carry over from the previous season and on crop demand did not affect the total wheat and maize grain yields relative to the farmers’ N rate in the rotation system, but improved N efficiency and net income, while decreasing the production cost and apparent N loss into the environment. An overall reduction of the N rate by 37–39% compared to farmers’ N, together with different control measures, using DCD was the most effective N control measure that increased yields, enhanced N utilization and economic benefit, while mitigating N loss.

## Author Contributions

ZP design and direct the experiment, also write this paper. YLiu, YLi, YW, and MM do the experiment, sample soil and plant, determine samples of soil and plant, deal with data, make tables and figures. YA and D-AA-V give the guidance of experiment design and revise the English language.

## Conflict of Interest Statement

The authors declare that the research was conducted in the absence of any commercial or financial relationships that could be construed as a potential conflict of interest.
